# Magnesium Phthalocyanines and Tetrapyrazinoporphyrazines: The Influence of a Solvent and a Delivery System on a Dissociation of Central Metal in Acidic Media

**DOI:** 10.3390/ph15040409

**Published:** 2022-03-27

**Authors:** Michaela Kolarova, Anita Mulaku, Miroslav Miletin, Veronika Novakova, Petr Zimcik

**Affiliations:** Department of Pharmaceutical Chemistry and Pharmaceutical Analysis, Faculty of Pharmacy in Hradec Kralove, Charles University, Akademika Heyrovskeho 1203, 500 05 Hradec Kralove, Czech Republic; michaela.kolarova@faf.cuni.cz (M.K.); anitamulaku@gmail.com (A.M.); miletin@faf.cuni.cz (M.M.); veronika.novakova@faf.cuni.cz (V.N.)

**Keywords:** magnesium phthalocyanine, dissociation, liposomes

## Abstract

Magnesium complexes of phthalocyanines (Pcs) and their aza-analogues have a great potential in medical applications or fluorescence detection. They are known to demetallate to metal-free ligands in acidic environments, however, detailed investigation of this process and its possible prevention is lacking. In this work, a conversion of lipophilic and water-soluble magnesium complexes of Pcs and tetrapyrazinoporphyrazines (TPyzPzs) to metal-free ligands was studied in relation to the acidity of the environment (organic solvent, water) including the investigation of the role of delivery systems (microemulsion or liposomes) in improvement in their acido-stability. The mechanism of the demetallation in organic solvents was based on an acidoprotolytic mechanism with the protonation of the azomethine nitrogen as the first step and a subsequent conversion to non-protonated metal-free ligands. In water, the mechanism seemed to be solvoprotolytic without any protonated intermediate. The water-soluble magnesium complexes were stable in a buffer with a physiological pH 7.4 while a time-dependent demetallation was observed in acidic pH. The demetallation was immediate at pH < 2 while the full conversion to metal-free ligand was done within 10 min and 45 min for TPyzPzs at pH 3 and pH 4, respectively. Incorporation of lipophilic magnesium complexes into microemulsion or liposomes substantially decreased the rate of the demetallation with the latter delivery system being much more efficient in the protection from the acidic environment. A comparison of two different macrocyclic cores revealed significantly higher kinetic inertness of magnesium TPyzPz complexes than their Pc analogues.

## 1. Introduction

Magnesium phthalocyanines (Pcs), similarly to their various aza-analogues and lower homologues, have attracted the attention of researchers since their first reports by Linstead in the 1930s [1,2]. Their synthesis is simple and they are readily accessible by the reaction of suitable substituted dinitriles with magnesium alkoxides [3,4,5]. They were investigated in several applications, e.g., for panchromatic light-harvesting, [6] for the formation of Langmuir–Shaefer films, [7] or as coatings for electrodes by porphyrazine/multiwalled carbon nanotube hybrids. [8] Magnesium is a light atom and therefore its Pc and porphyrazine complexes are often characterized by a strong fluorescence that is utilized in the development of fluorescent sensors [9,10,11]. The magnesium complexes of Pcs and porphyrazines are also able to efficiently produce a singlet oxygen and for this reason they have been tested several times in photodynamic applications in the treatment of cancer [12,13,14] or bacterial infections [15,16,17,18]. If required, the singlet oxygen production of magnesium complexes in these medical applications can be further increased by the peripheral substitution of heavy-atoms [19,20]. The central magnesium cation in Pcs and their analogues is, however, not very stable and can be removed using various acids [21,22,23]. This is advantageously utilized in the synthesis of metal-free ligands that are firstly prepared using magnesium alkoxide-assisted cyclotetramerization and subsequently demetallated [24,25]. On the other hand, the low resistance of magnesium complexes against acids and easy dissociation to metal-free derivatives in acidic media may be undesirable properties if the intended compounds for various applications are magnesium derivatives. The demetallation may have unexpected consequence since the compounds are often tested under acidic conditions (e.g., cancer tissues have an acidic pH or lysosomes (a typical target of many hydrophilic PSs) also have a pH value of around 5) without any care about the stability of the central magnesium cation. As efficient photosensitizers and/or diagnostic fluorescence imaging tools, Pc and TPyzPzs can meet acidic environments in vivo, typically in some cancer tissues and inflamed areas. Also, in the case of development of targeted third generation photosensitizers by conjugation of a Pc/TPyzPzs with monoclonal antibodies, specific oligonucleotides (antisense or aptamers) or other targeting tools (e.g., liver cells targeting *N*-acetylgalactosamine) will enter the cells via endosomes or lysosomes. Therefore, the detailed knowledge about their behavior in various pH conditions is crucial to properly design efficient molecules. The unintentionally arisen metal-free ligands of Pcs and porphyrazines often suffer from a low fluorescence or singlet oxygen quantum yields that completely change their usability in photodynamic application or in fluorescence sensing [10,26].

The aim of this project was, therefore, to evaluate and discuss the kinetic inertness of the magnesium complexes of Pc and its aza-analogue of tetrapyrazinoporphyrazine (TPyzPz) type in various acidic environments. Besides water and organic solvent, microemulsion and liposomes were selected for testing as well since both were recently applied as delivery systems in several studies and the access of acid may be potentially restricted by the lipophilic environment [11,19,27,28].

## 2. Results and Discussion

### 2.1. Design of the Study

**Figure 1:**
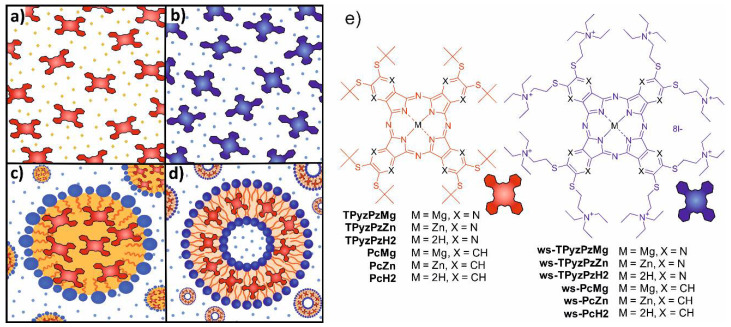
Schematic illustration of the studied systems: (**a**) organic solvent (benzene) with the dissolved lipophilic dyes; (**b**) water with the dissolved hydrophilic dyes; (**c**) microemulsion with the lipophilic dyes incorporated in the oil particles; (**d**) liposomes with the lipophilic dyes incorporated in the lipidic bilayer; and (**e**) structures of the concerned compounds shown on the right.

The study was focused on two types of Pcs (lipophilic and hydrophilic) and their aza-analogues TPyzPzs (Figure 1). Both of these structural types have been previously extensively studied in photodynamic applications or fluorescence sensing [11,12]. The magnesium complexes **PcMg** and **TPyzPzMg** are highly lipophilic with bulky peripheral substituents to decrease the aggregation and were selected for the study in organic solvent (benzene) and in the lipophilic delivery systems of microemulsions and liposomes (Figure 1a,c,d). The water-soluble analogues **ws-PcMg** and **ws-TPyzPzMg** are substituted by charged cationic substituents that ensure a high water-solubility (Figure 1b). The electronic effects of alkylsulfanyl substituents in all studied compounds (lipophilic and hydrophilic) are the same and thus the influences of different environments (solvent, delivery systems or water) can be easily studied without being affected by the electron density of the macrocyclic ligand.

In the case of water and benzene, the zinc analogues were studied as well to allow a comparison with a macrocycle which is not demetallated under acidic conditions and to distinguish between a demetallation and other possible processes (e.g., a protonation of the porphyrazine core).

The stability under acidic conditions was monitored spectroscopically by observing changes in the Q-band area of the absorption spectra. The metal complexes (both Mg and Zn) are characterized by the sharp and unsplit Q-band. On the other hand, the produced metal-free derivatives possess the split Q-band due to the lower *D*_2h_ symmetry. 

### 2.2. Organic Solvent

**Figure 2:**
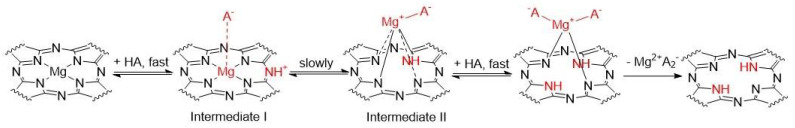
The acidoprotolytic mechanism of dissociation of Mg complexes in organic solvent in presence of acid HA. Based on ref. [29].

Several works have suggested that the process of the dissociation of magnesium Pcs and analogues in organic solvents involves several intermediates (some of them appearing only very shortly) depending on the type of solvent used. In benzene, they suggested an acidoprotolytic mechanism where the acid acts as a proton donor and the anion at the same time [29,30]. As the first typical step of this mechanism, a protonation of the azomethine nitrogen (Figure 2, Intermediate I) and an anion coordination to central magnesium were suggested.

**Figure 3:**
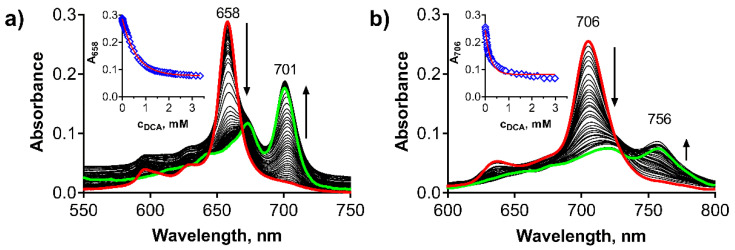
Changes in absorption spectra during the protonation of (**a**) **TPyzPzMg** and (**b**) **PcMg** in benzene (*c*_dye_ = 1 μM) after the addition of DCA. Insets: Changes of the absorbance of the main absorption Q-band of the non-protonated form, red line = nonlinear fit. All data were corrected for a dilution.

To confirm this mechanism, we examined the protonation of the macrocycles leading to Intermediate I first. A stepwise addition of dichloroacetic acid (DCA) into benzene solution of **PcMg** and **TPyzPzMg** led to a decrease in the intensity of the Q-band with an appearance of the novel, red-shifted band at approximately 756 nm and 701 nm, respectively (Figure 3). Based on the literature data, this band can be attributed to the formation of the monoprotonated form of the macrocycle (**PcMgH^+^**, **TPyzPzMgH^+^**) on azomethine nitrogen (Figure 2, Intermediate I) [31,32,33,34]. The association constant of the macrocycle and DCA in benzene were determined from the titration data (Figure 3, insets) to be *K*_a_ = 4.33 ± 0.31 × 10^3^ M^−1^ and 1.67 ± 0.026 × 10^3^ M^−1^ for **PcMg** and **TPyzPzMg**, respectively, indicating a lower basicity of the latter macrocycle due to the presence of electron-withdrawing pyrazine rings. When the titration of **TPyzPzMg** was performed with more acidic trifluoroacetic acid (TFA, Figure S1), the *K*_a_ value increased to 4.24 ± 0.20 × 10^4^ M^−1^.

Similar spectral changes (Figure S2) after the addition of DCA were observed also for the corresponding zinc complexes confirming that the protonation of an azomethine nitrogen is taking place in acidic environments irrespective of the central metal. Their *K*_a_ values were, however, considerably lower than for the corresponding magnesium complexes (1.17 ± 0.02 × 10^3^ M^−1^ and 3.47 ± 0.05 × 10^2^ M^−1^, for **TPyzPzZn** and **PcZn**, respectively). This finding is in line with observations of the increased basicity of magnesium Pcs in comparison with other metal complexes which have been already noted in the literature [35].

**Figure 4:**
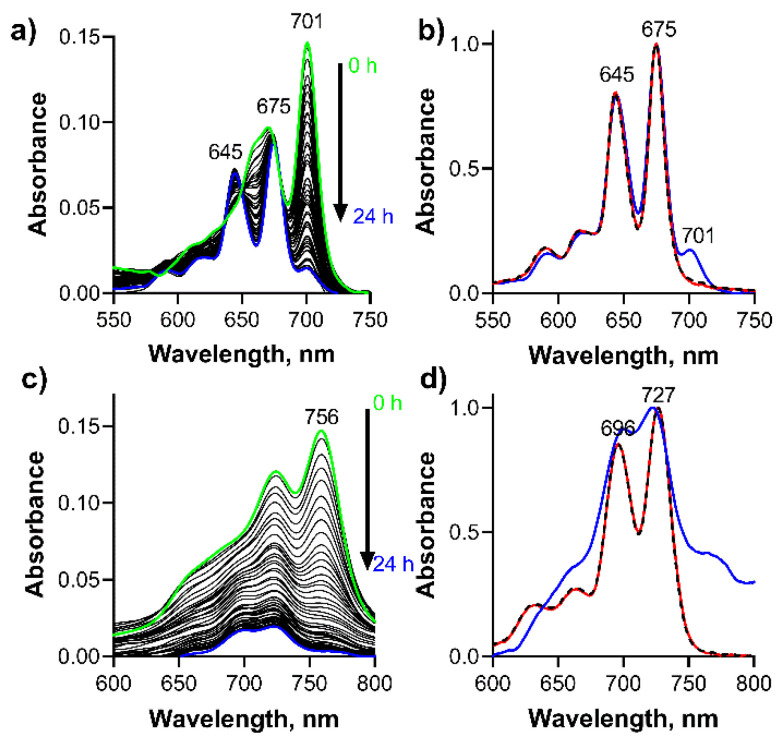
Changes of the absorption spectra of protonated (green line) (**a**) **TPyzPzMgH**^+^ and (**c**) **PcMgH**^+^ (*c*_dye_ = 1 µM) during 24 h in solution of DCA (*c*_DCA_ = 4.75 mM) in benzene at 25 °C. The normalized absorption spectra of (**b**) TPyzPzMg, (**d**) **PcMg** demetallated by DCA (blue line) in benzene (*c*_DCA_ = 4.75 mM, 24 h treatment, data from boxes (**a**,**c**)). For comparison, the spectra of (**b**) **TPyzPzH2** and (**d**) **PcH2** in benzene (red) and benzene with DCA (*c*_DCA_ = 4.75 mM, black dashed) are also shown indicating no protonation of metal-free ligands.

While no qualitative changes were observed for the monoprotonated zinc complexes in time even after 24 h (besides a slight decomposition of **PcZn** indicated by overall decrease of the absorption at all wavelengths, Figure S2f) due to known high kinetic inertness of these complexes, the spectra of the protonated magnesium macrocycles evolved in time. The spectrum of monoprotonated **TPyzPzMgH^+^** (Intermediate I) was replaced by a new spectrum with the split Q-band and the blue-shifted maxima at 675 nm and 645 nm (Figure 4a) with clear isosbestic points indicating a transformation of one species to another. The change was irreversible. The shape of the spectrum after 24 h of treatment with 4.75 mM DCA perfectly overlapped the spectrum of metal-free **TPyzPzH2** (the last structure on Figure 2) with only a small residue of non-demetallated **TPyzPzMgH^+^** appearing as the shoulder at 701 nm (Figure 4a,b). This further supported the mechanism suggested in Figure 2. The fact that the final product of this reaction, **TPyzPzH2**, is in non-protonated form, has been confirmed by dissolving **TPyzPzH2** in benzene and after the addition of DCA (c = 4.75 mM, Figure 4b). The spectra overlapped each other indicating no protonation at this acid concentration which is again in agreement with the reported very low basicity of metal-free Pcs and TPyzPzs [31]. In the case of the demetallation of **PcMgH^+^**, similar changes were observed as for the pyrazine analogue, but they were accompanied by its decomposition as deduced from an overall decrease of the absorption intensity at all wavelengths with no apparent isosbestic point (Figure 4c). Despite this, the final product after 24 h was characterized by the absorption maxima in the same position as for metal-free **PcH2**, similarly as in the case of the pyrazine analogue, indicating that also in this case, the final product is a non-protonated metal-free ligand as suggested in Figure 2. 

**Figure 5:**
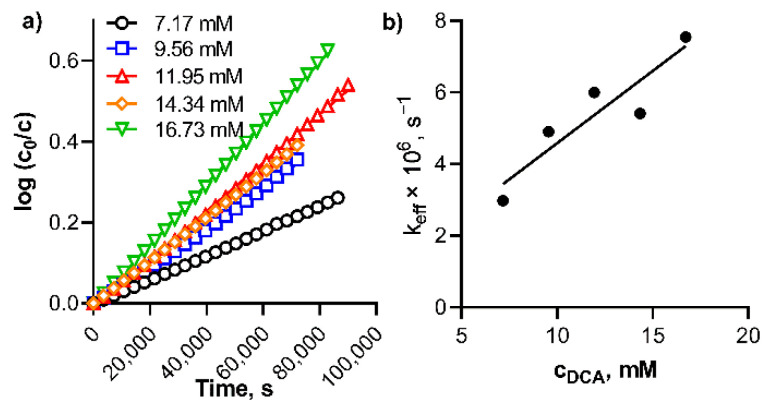
(**a**) Plot of log(*c*_0_/*c*) against time for the dissociation of **TPyzPzMgH^+^** in benzene at different concentrations of DCA at 20 °C, and (**b**) Plot of effective rate constant, *k*_eff_, (calculated from the data in box (**a**)) versus the concentration of DCA.

The kinetic data from Figure 4 were used to calculate the rate of the dissociation of **TPyzPzMgH^+^** in benzene with DCA. It followed the first order kinetic (Figure 5a). The effective rate constants, *k*_eff_, for the dissociation increased linearly with increasing DCA concentrations (Figure 5b) in a row 2.98 × 10^−6^, 4.90 × 10^−6^, 6.00 × 10^−6^, 5.41 × 10^−6^ and 7.54 × 10^−6^ s^−1^ for the DCA concentrations 7.17, 9.56, 11.95, 14.34 and 16.73 mM, respectively. From these data, the true rate constant *k*_V_ = *k*_eff_/*c*_DCA_ was calculated to be *k*_V_ = 4.52 ± 0.57 × 10^−4^ s^−1^ M^−1^_._ Also, the kinetic data therefore supported the acidoprotolytic mechanism suggested in the literature for this type of dissociation [30]. Due to the decomposition observed during the dissociation of **PcMgH^+^**, the kinetic data could not be properly analyzed for this compound.

The experiments in benzene, besides confirming the suggested mechanism of the acidoprotolytic dissociation, [30] led to further conclusions about the basicity of azomethine nitrogens, the point of the molecule where the dissociation of magnesium complexes starts. Firstly, the basicity of azomethine nitrogens in magnesium complexes of Pcs and TPyzPzs is substantially increased over zinc complexes and, in particular, over metal-free ligand. Second, the basicity of **MgTPyzPz** core was lower than that of **PcMg**.

### 2.3. Water

**Figure 6:**
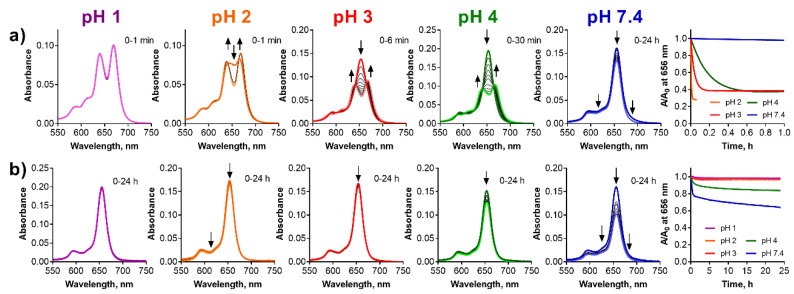
Changes of the absorption spectra of (**a**) **ws-TPyzPzMg** and (**b**) **ws-TPyzPzZn** at different pH values of the buffer (*c*_dye_ = 1 µM). The utmost right graphs show normalized decrease of the absorbance at the *λ*_max_ of the parent magnesium complexes with time.

The behavior of Pcs and TPyzPzs was studied in Britton–Robinson buffers at acidic pH values (1–4) as well as at a physiological pH = 7.4. First, **ws-TPyzPzZn**, which is monomeric and kinetically inert in this buffer, was investigated. No changes were observed within 24 h at pH < 3 while at pH = 4, and even more significantly at pH = 7.4, a decrease in the absorbance at all wavelengths was detected without the change of the shape of the spectra (Figure 6b). This behavior can be attributed to a well-known phenomenon of an adsorption of cationic compounds to the negatively charged glass surface [36,37]. The silanol groups on the glass surface are acidic (several different silanol groups are present, the most acidic has p*K*_a_ = 4.8) that introduces the negative charge to the surface at pH > 4 [38]. On the other hand, the charge density of glass is almost zero in the acidic environment at pH < 4 [39]. The results of our study with limited adsorption of cationic **ws-TPyzPzZn** at pH < 3 are in full agreement with these findings. To further support this explanation of the decreased absorbance, **ws-TPyzPzZn** in buffer of pH = 7.4 was left in a cuvette for 24 h; the cuvette was emptied; carefully washed three times with water and filled with distilled water to the same volume as used during the investigation. The absorption spectrum indicated that the cationic compound adsorbed to the glass surface was released back to the solution (Figure S3). 

Similarly, to the zinc complex, **ws-TPyzPzMg** was also adsorbed to the glass at pH 7.4 without any qualitative changes observed in the spectrum indicating a full stability during the studied period of 24 h (Figure 6a). Lowering of the pH led to the demetallation, which was detected by a typical split of the Q-band of its product, **ws-TPyzPzH2**. The rate of the demetallation was also a function of time; the lower pH the faster demetallation. The demetallation was finished almost immediately at pH 1 after the adding of the sample to the buffer and potential residuals of the magnesium complex were not even detected. The residuals of the magnesium complex were observed at pH 2 just after the addition of the sample to the solution. The demetallation was finished in 10 min and in 45 min at pH 3 and pH 4, respectively. Contrary to the above observations in organic solvents, the azomethine-protonated form, **ws-TPyzPzMgH^+^**, was not observed at any pH indicating that the mechanism of the demetallation might be different in water, most likely solvoprotolytic as suggested by Berezin et al. for a polar DMSO/H_2_SO_4_ system [29]. In this mechanism, the magnesium complex is directly converted to metal-free ligand without any intermediates similarly as observed in our experiments.

**Figure 7:**
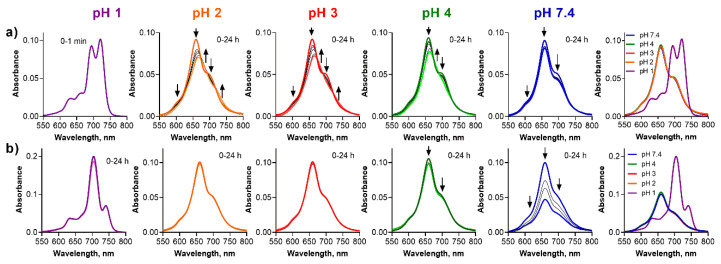
Changes of the absorption spectra of (**a**) **ws-PcMg** and (**b**) **ws-PcZn** at different pH values of the buffer (*c*_dye_ = 1 µM). The utmost right graphs show the absorption spectra just after addition of the stock solution of the dye to the buffer.

The investigation of the Pc analogues **ws-PcMg** and **ws-PcZn** was hampered by their clearly aggregated nature in the buffer at a pH above 2 (Figure 7, the utmost right graph). Despite this, the behavior of **ws-PcZn** was similar to the TPyzPz analogue. Strong adsorption of the cationic compound to glass surface was observed at pH 7.4 and slightly lower at pH 4. Almost no changes were detected at pH 2 and pH 3 (Figure 7b) for **ws-PcZn**. Surprisingly, a clearly monomeric spectrum was observed at pH 1 with appearance of a typical, red-shifted band corresponding to the small amount of azomethine-protonated species. 

For **ws-PcMg**, the adsorption to the glass surface was clear at pH 7.4 without any qualitative changes to the absorption spectra suggesting a full stability (Figure 7a). The absorption spectra changed the shape with time at pH 2–4 suggesting a slight demetallation process, but this could not be unequivocally confirmed and analyzed due to a strongly aggregated nature of the spectra. However, the data suggested somewhat slower progress of the demetallation in a comparison to the corresponding TPyzPz (e.g., the demetallation of **ws-TPyzPzMg** was finished within 10 min while the progress was still observable even after 24 h for **ws-PcMg**). A plausible explanation might be that the aggregated species are less prone to the attack of the acid due to a restricted access of protons to the aggregates. Further, **ws-PcMg** was demetallated to **ws-PcH2** at pH 1 immediately after the mixing of the solutions. At this pH, **ws-PcH2** is monomeric and non-protonated confirming further the lower basicity of metal-free ligands.

### 2.4. Microemulsion

**Figure 8:**
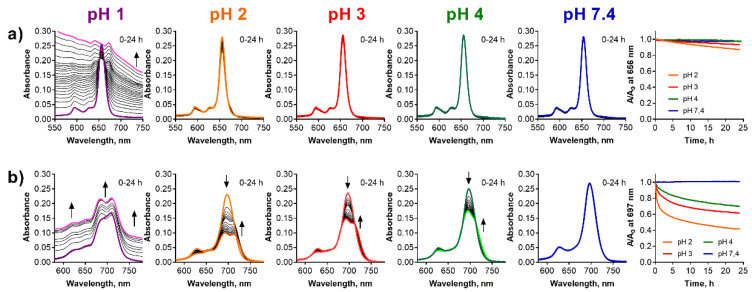
Changes of the absorption spectra of (**a**) **TPyzPzMg** and (**b**) **PcMg** in the microemulsion (*c*_dye_ = 1 µM) at different pH values of the buffer. The increasing backgrounds at pH = 1 are due to instability of the microemulsion in strongly acidic pH. The utmost right graphs show the normalized decrease of the absorbance at the *λ*_max_ of the parent magnesium complexes with time.

Ethoxylated castor oil/medium chain triglycerides in water microemulsions were employed as simple models of pegylated lipid nanoparticles, which are popular delivery systems for sensitive or purely water soluble pharmaceuticals. Microemulsions are clear, thermodynamically stable systems formed usually by oil particles stabilized in water by an addition of a suitable surfactant (see Figure 1c). The particles have hydrophobic core which may dissolve the lipophilic molecules, in our case the lipophilic **PcMg** and **TPyzPzMg**. Here, **TPyzPzMg** appeared to be fully stable in microemulsion at pH > 4 for at least 24 h. Only a small decrease in the absorbance of the Q-band due to demetallation was observed at pH 2 and 3 (Figure 8a). The data obtained at pH 1 could not be properly analyzed since this pH apparently led to the destruction of the delivery system as suggested by an increasing background during the measurements. Once the delivery system was destroyed, **TPyzPzMg** either precipitated from the solution or was destructed as indicated by the featureless absorption spectra in the Q-band area. The demetallation can potentially also take place during the destruction of microemulsion at pH 1, but this cannot be unequivocally confirmed or refuted from the obtained data. Contrary to the TPyzPz analogue, **PcMg** is stable in the microemulsion only at pH 7.4. At a lower pH, the changes in the absorption spectra indicated the demetallation process to **PcH2** with the pH-dependent rate (Figure 8b). Similarly, pH 1 destroyed the microemulsion but **PcMg** was converted almost immediately to **PcH2**. The mechanism of the demetallation in microemulsions is most likely also solvoprotolytic since no protonated species were detected during the measurements and the conversion ran directly from the magnesium complex to the metal-free ligand. A direct comparison of the two macrocycles (**PcMg** and **TPyzPzMg**) clearly indicated much higher stability of the **TPyzPzMg** in the microemulsion.

### 2.5. Liposomes

**Figure 9:**
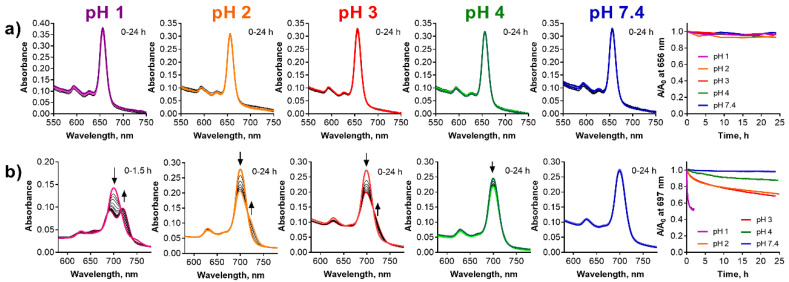
Changes of absorption spectra of (**a**) **TPyzPzMg** and (**b**) **PcMg** in the liposomes (*c*_dye_ = 1 µM) at different pH values of the buffer. The increased background is due to the light-scattering in the liposomal suspension. The utmost right graphs show the normalized decrease of the absorbance at the *λ*_max_ of the parent magnesium complexes with time.

Liposomes are spherical-shaped vesicles formed by a lipophilic bilayer with a hydrophilic inner compartment (see Figure 1d). The lipophilic bilayer may host lipophilic compounds and, in addition to functioning as a delivery system or drug carrier, it can also serve as a simple model of biomembranes [40,41]. Contrary to the microemulsion, the liposomes as a delivery system were stable also at pH 1. The observation of the absorption spectra of **TPyzPzMg** in the liposomes did not indicate any change at any pH even after 24 h of the treatment and the compound in the liposomes was therefore fully protected against the attack of the acidic environment (Figure 9a). Similarly, **PcMg** in the liposomes appeared to be less stable in this delivery system than the TPyzPz analogue (Figure 9b). The compound was fully stable for 24 h only at a physiological pH 7.4. The decrease of the absorption at the Q-band at pH 4 and the typical increase of the absorption of **PcH2** at longer wavelengths at pH 2 and pH 3 during 24 h suggested that the process of the demetallation occurred. This was confirmed by the full demetallation at pH 1 within 1.5 h of the treatment. Data from both **PcMg** and **TPyzPzMg** in liposomes can be compared also with the previous delivery system. The comparison of the results for both compounds clearly indicated a better protection of incorporated lipophilic compounds from the acidic environment in liposomes than in microemulsion.

## 3. Materials and Methods

### 3.1. General

All studied Pcs and TPyzPzs have been already prepared and published by our research group [10,12,42]. All organic solvents and other chemicals used for synthesis and spectral measurements were of analytical grade. The UV/Vis spectra were recorded using Shimadzu UV-2600 spectrophotometer at 20 °C or 25 °C (controlled by a Peltier cooled cuvette holder). All data were processed with UV Probe 2.42 software. The experiments in organic solvents were performed with anhydrous benzene (Sigma-Aldrich, St. Louis, MO, USA, water content <0.001%) with dichloroacetic acid (DCA, Carlo-Erba) or trifluoroacetic acid (TFA, Fluorochem). The Britton–Robinson buffer (containing 0.04 M H_3_BO_3_, 0.04 M H_3_PO_4_, 0.04 M CH_3_COOH—total volume 120 mL) was adjusted to desired pH values (7.4, 4.0, 3.0 and 2.0) by mixing with appropriate volume of 0.2 M NaOH (66; 23; 20; 6 mL, respectively). For the buffer of pH 1.0, an addition of H_3_PO_4_ and 98% acetic acid was used. The pH was measured using a pH 8+ DHS pH meter (XS Instruments) calibrated with a two-point calibration (pH 7.00 and 4.00) prior to use.

### 3.2. Lipophilic Samples in Organic Solvent

An anhydrous benzene solution (1 μM) of the lipophilic compounds (**TPyzPzMg**, **TPyzPzZn**, **PcMg**, **PcZn, TPyzPzH2** and **PcH2**) was prepared and 2 mL were transferred into a quartz cuvette. For titration experiments, a specific volume of trifluoroacetic (TFA) or dichloroacetic (DCA) acid solutions in anh. benzene had been added. The absorption spectra were recorded immediately after every single addition of TFA or DCA. For the determination of *k*_eff_, 2.49 mL of a prepared stock solution in benzene (1 μM) in a quartz cuvette was mixed with 10 μL of a prepared DCA solution in anh. benzene (final concentration of DCA in the cuvette was 7.17 mM, 9.56 mM, 11.95 mM, 14.34 mM and 16.73 mM); and the absorption spectra were recorded in a 24 h time-lapse sequence with time interval 1 h in the cuvette holder tempered at 20 °C. The effective rate constant, *k*_eff_, for a dissociation of **MgTPyzPzH^+^** to **TPyzPzH2** in benzene/DCA, was determined from the linear regression of the dependence of the log(*c*_0_/*c*) on the time *t*, where *c*_0_ and *c* are the concentrations of protonated **MgTPyzPzH^+^** in the beginning and in the time *t*, respectively. All spectra presented in the article were corrected for a dilution. 

### 3.3. Water-Soluble Samples in Buffers

A water stock solution (100 μM) of the water-soluble compounds (**ws-TPyzPzMg**, **ws-TPyzPzZn**, **ws-PcMg** and **ws-PcZn**) was prepared and 25 μL of this stock solution was added to the tested buffer solution (2.48 mL) in a quartz cuvette to prepare a tested solution at a concentration of 1 μM. The absorption spectra were recorded immediately after the mixing and then in the selected time intervals (intervals were set between 30 s to 30 min depending on the speed of the process) typically up to 24 h.

### 3.4. Preparation of Microemulsions and Evaluation of Stability of the Complexes

A THF stock solution (100 μM) of lipophilic compounds **TPyzPzMg** or **PcMg** was prepared and a volume of 1.25 mL of this solution was added to a mixture of polyoxyl 35 hydrogenated castor oil (Cremophor^®^ EL, BASF, Ludwigshafen, Germany) (72 mg) and medium-chain triacylglycerides (29 mg) (Ecogreen oleochemicals, meeting specifications of Ph. Eur.) dissolved in 4 mL of chloroform. The solvent was evaporated in a water bath tempered at 40 °C under reduced pressure and kept under the pressure of 5 mbar for 30 min to remove all residuals of solvents. Subsequently, the Britton–Robinson buffer of pH 7.40 (4 mL) was added and the mixture was vortexed for 5 min. After homogenization, the volume was adjusted to 5 mL in a volumetric flask. Prepared microemulsion (“stock microemulsion”) contained the dye at concentration of 25 μM incorporated in the oil particles. Microemulsions of such compositions have already been characterized using dynamic light scattering with the approximate size of the particles approximately 300 nm [43].

During the following experiment, the stock microemulsion (100 μL) was added to the tested buffer solution (2.4 mL) in a quartz cuvette to prepare the tested mixture at the final concentration of 1 μM. The absorption spectra were recorded immediately after mixing and then within 24 h in suitable time intervals (2–30 min) depending on the rate of the demetallation process.

### 3.5. Preparation of Liposomes and Evaluation of Stability of the Complexes

Large unilamellar vesicles formed by an extrusion technique (LUVETs) were prepared from a suspension of multilamellar vesicles (MLVs) using the following procedure. Dioleoylphosphatidylcholine (DOPC, 19.7 mg, 25 μmol) (Lipoid GmbH, Germany) was dissolved in chloroform and a THF stock solution (0.25 mL, 100 μM) of **TPyzPzMg** or **PcMg** was added. The solvents were evaporated in a 100 mL round-bottom flask on a water bath tempered at 37 °C and kept on the water bath for 30 min under the pressure of 5 mbar to remove all residues of organic solvents. The Britton–Robinson buffer of pH 7.4 (1.0 mL) was added and the lipids were removed from the flask walls by a gentle hand shaking. The prepared suspension was vortexed for 5 min to form MLVs and was left for 24 h at room temperature to complete the swelling. Subsequently, using a small hand extruder (LiposoFast Basic, Avestin), the suspension was passed back and forth 21 times through two stacked polycarbonate filters (pore size 100 nm) at room temperature. The final suspension of liposomes (“stock liposomal suspension”) contained the dye at a concentration of 25 μM incorporated in the unilamellar DOPC vesicles with a lipid concentration of 25 mM, i.e., with dye-to-lipid ratio 1:1000. Liposomes of such composition have already been characterized in our laboratory using a dynamic light scattering with the approximate size about 128 nm [44].

Subsequently, the stock liposomal suspension (100 μL) was added to the tested buffer solution (2.4 mL) in a quartz cuvette to prepare a tested mixture at the final concentration of dyes 1 μM. The absorption spectra were recorded immediately and then within 24 h with the time intervals selected according to the speed of changes from 2 min to 30 min.

## 4. Conclusions

The aim of the study was to determine the stability of the magnesium complexes of TPyzPzs and Pcs in different environments under acidic conditions. In organic solvents, the data suggested that basic azomethine nitrogens in the TPyzPz and Pc core are protonated after the addition of acid with a subsequent demetallation to non-protonated metal-free ligands, which confirmed the acidoprotolytic mechanism of the demetallation process in organic solvents. [29] However, the demetallation in water seems to proceed by a solvoprotolytic mechanism where the azomethine-protonated form was not detected and the magnesium derivative is converted directly to a metal-free ligand.

The demetallation of **ws-TPyzPzMg** in water was taking place already at pH 4 and was faster in a more acidic pH. Introduction of the lipophilic Pcs and TPyzPzs into delivery systems (microemulsions, liposomes) protected them substantially from the attack by the acidic environment. For example, while **ws-TPyzPzMg** in buffer was fully demetallated within few minutes at pH 2, the absorbance of lipophilic analogue **TPyzPzMg** at the same pH decreased only for approximately 10% within 24 h in the microemulsion and once incorporated into the liposomes the compound did not show any detectable changes at pH 2 for the same period of time. The data also indicated that liposomes offer a better protection against the acidity for magnesium complexes than the microemulsion system. The direct comparison between Pc and TPyzPz core could be carried out only for the delivery systems. Nevertheless, the data in both, the liposomes and the microemulsion, proved that the magnesium complexes of TPyzPz derivatives were more stable.

Findings from this study may lead to important consequences for the use of magnesium complexes of Pcs and TPyzPzs in in vitro or in vivo assays where a lower pH must be considered (for example, the acidic organelles lysosomes typically targeted by potential photosensitizers have a pH of 4–5 [45]). Once incorporated into lipidic bilayers, the risk of demetallation of lipophilic magnesium complexes is negligible at a physiologically achievable pH as suggested from the data in liposomes, the models of biological membranes, and the compounds may be considered as stable. Additionally, potential pH-sensing using magnesium TPyzPzs incorporated into liposomes (e.g., in a recent publication of pH-sensors [11]) should be achievable even at a much lower pH without any problems with stability of the central cation. At the same time, the microemulsions as a delivery system also proved to protect the lipophilic complexes sufficiently from low pH and therefore can be used in cellular systems without any limitations. On the other hand, well water-soluble and non-aggregating magnesium complexes of Pcs and TPyzPzs in cells are found in water environments in a lysosomal matrix. In this case, they are demetallated to the metal-free derivatives (in particular, at longer incubation times) and subsequently the tested compounds (e.g., for photodynamic activity or toxicity) are of the metal-free form and not the originally applied magnesium complexes. This consequence should be considered during the evaluation of the in vitro results.

## Data Availability

Data is contained within the article and Supplementary Material.

## Supplementary Materials

The following supporting information can be downloaded at: https://www.mdpi.com/article/10.3390/ph15040409/s1, Figure S1: Protonation of **TPyzPzMg** in benzene by TFA; Figure S2: Protonation of **TPyzPzZn** and **PcZn** in benzene by DCA; Figure S3: Adsorption of **ws-TPyzPzZn** on the glass surface of cuvette. Figure S4: Absorption spectra of lipohilic compounds. Figure S5: Absorption spectra of water-soluble compounds. Analytical data for the studied compounds from literature. Reference [46] is cited in Supplementary Material.
