# Supplementary material for: Magnesium Phthalocyanines and Tetrapyrazinoporphyrazines: The Influence of a Solvent and a Delivery System on a Dissociation of Central Metal in Acidic Media
Source: Pharmaceuticals (Basel). 2022 Mar 27;15(4):409. doi: 10.3390/ph15040409 (PMC9027660; doi:10.3390/ph15040409)
Supplement: Supplementary file 1 [file pharmaceuticals-15-00409-s001.zip › pharmaceuticals-1615067-supplementary.pdf]

Supplementary Materials

Magnesium phthalocyanines and tetrapyrazinoporphyrazines: the influence of a solvent and a delivery system on a dissociation of central metal in acidic media

Michaela Kolarova, Anita Mulaku, Miroslav Miletin, Veronika Novakova, Petr Zimcik*

Department of Pharmaceutical Chemistry and Pharmaceutical Analysis, Faculty of Pharmacy in Hradec Kralove, Charles University, Akademika Heyrovskeho 1203, Hradec Kralove, 500 05, Czech Republic.

* Correspondence: zimcik@faf.cuni.cz; Tel.: +420 495067257

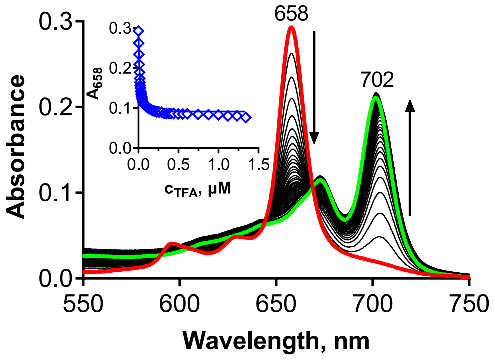

Figure S1. Protonation of **TPyzPzMg** in benzene (c_{dye} = 1 μM) after addition of TFA**.** Inset: Changes of absorbance of the main absorption Q-band of non-protonated form. All data were corrected for dilution.

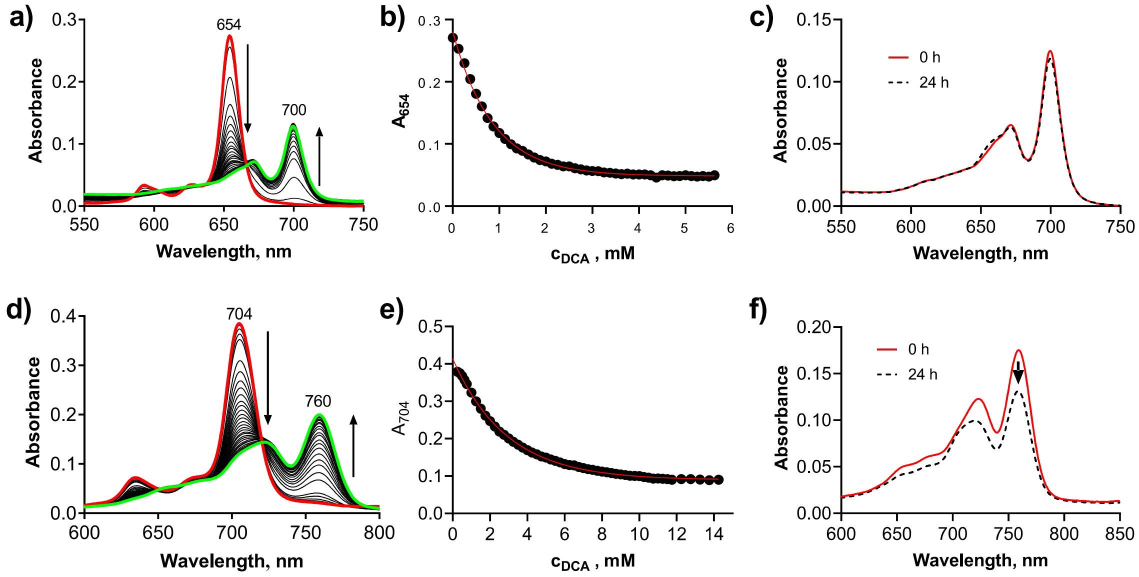

Figure S2. a, d) Changes in absorption spectra during protonation of **TPyzPzZn** (a) and **PcZn** (d) in benzene (c_{dye} = 1 μM) after addition of DCA. All data were corrected for dilution. b, e) Changes of absorbance of the main absorption Q-band of non-protonated form of **TPyzPzZn** (b) and **PcZn** (e), red line = nonlinear fit. c, f) Changes of the absorption spectra of the acidic solution of **TPyzPzZn** (c, c_{DCA}= 5.5 μM) and **PcZn** (f, c_{DCA}= 14.5 μM) in benzene at full protonation of azomethine nitrogen after 24 h.

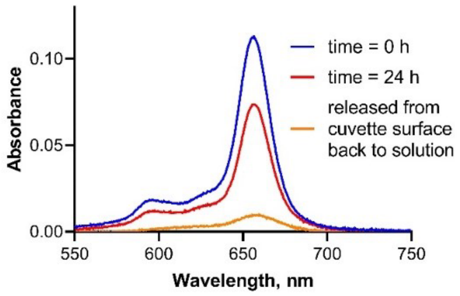

Figure S3. Absorption spectra of **ws-TPyzPzZn** in buffer at pH 7.4 just after mixing the solutions (blue), after 24 h (red) and after release of the sample from cuvette surface back to solution (cuvette was washed with distilled water three times and filled with distilled water to the original volume – orange).

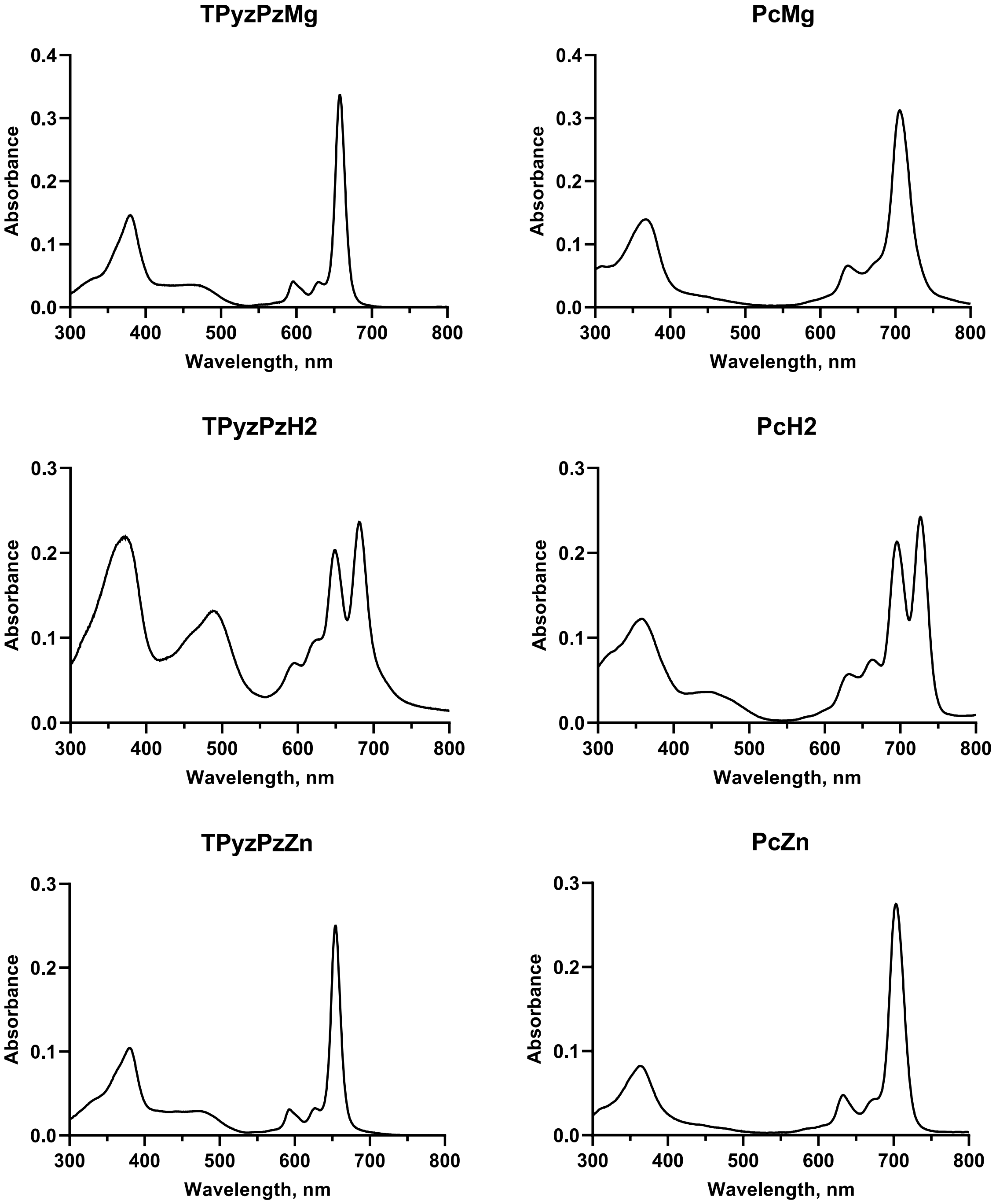

Figure S4. Absorption spectra of lipophilic compounds in benzene at c=1 μM.

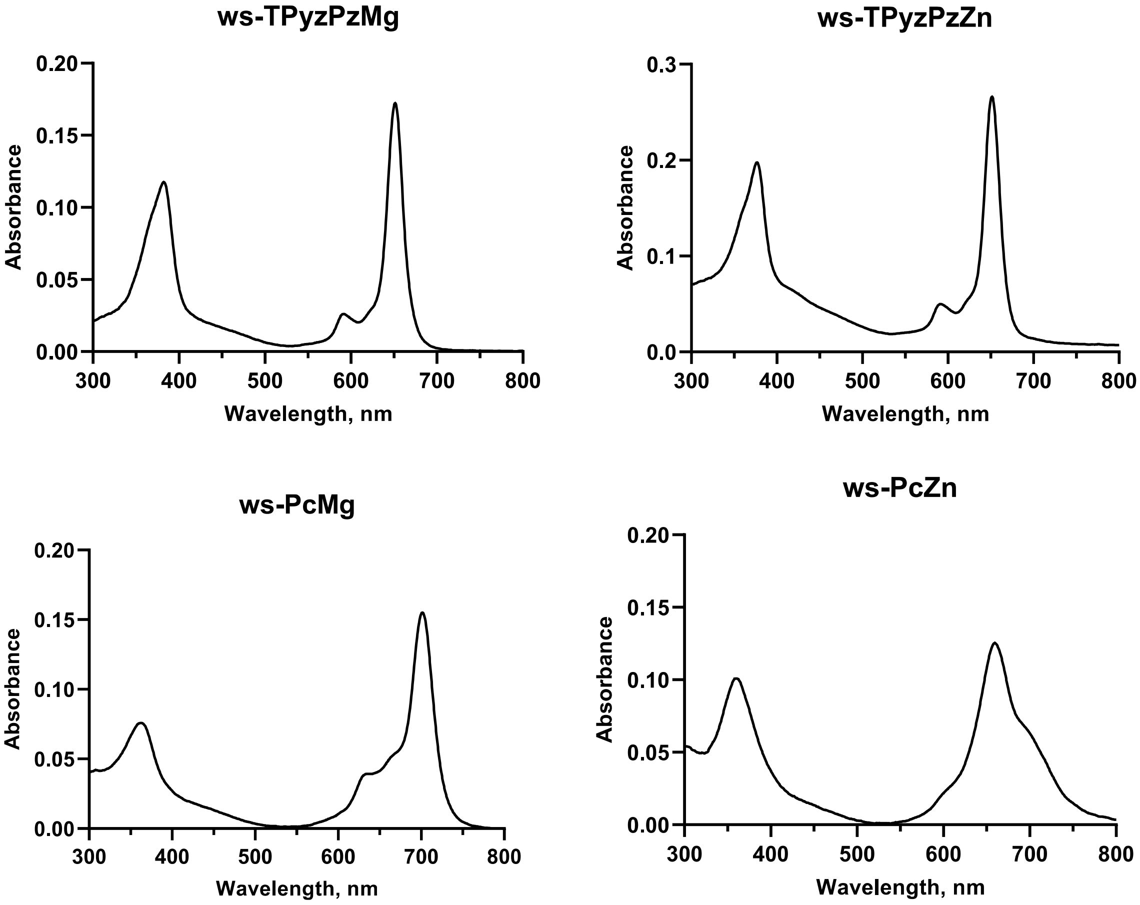

Figure S5. Absorption spectra of water-soluble compounds in water at c=1 μM.

Analytical Data for the Studied Compounds from Literature

2,3,9,10,16,17,23,24-Octakis(*tert*-butylsulfanyl)-1,4,8,11,15,18,22,25-(octaaza)phthalocyaninato Magnesium(II) (**TPyzPzMg**)

Rf (toluene/chloroform/THF 10:10:1) = 0.36. MS (MALDI-TOF) *m*/*z*: 1248.1 $[M]^+$, 1271.1 $[M^+Na]^+$, 1287.1 $[M^+K]^+$, 2496.3 $[2M]^+$. UV-vis (DMF) *λ*/nm (ε/$M^{-1}cm^{-1}$): 655 (281 200), 593 (37 700), 379 (147 700). UV-vis (THF) *λ*/nm (ε/$M^{-1}cm^{-1}$): 651 (335 800), 624 (40 000), 591 (42 100), 383 (166 800). Elemental analysis calculated for $C_{56}H_{72}MgN_{16}S_8$+3H_2O: C 51.57, H 6.03, N 17.18%. Found: C 51.49, H 5.86, N 16.84%. Data from ref. [1]

UV–vis (pyridine) *λ*/nm (ε/$M^{-1}cm^{-1}$): 658 (298 000), 596 (36 400), 382 (145 000). ^{13}C NMR (75.43 MHz, $CDCl_3$) δ/ppm = 30.6, 51.2, 144.2,151.1, 158.3. ^{1}H NMR (300 MHz, $CDCl_3$) δ/ppm = 1.94 (s, 72H, CH_3). Data from ref. [2]

2,3,9,10,16,17,23,24-Octakis(*tert*-butylsulfanyl)-1,4,8,11,15,18,22,25-(octaaza)phthalocyaninato Zinc(II) (**TPyzPzZn**)

Rf (toluene/chloroform/THF 10:10:1) = 0.65. MS (MALDI-TOF) *m*/*z*: 1288.1 $[M]^+$, 1311.1 $[M^+Na]^+$, 1327.1 $[M^+K]^+$. UV-vis (DMF) *λ*/nm (ε/$M^{-1}cm^{-1}$): 654 (268 500), 593 (35 700), 383 (137 300). Elemental analysis calculated for $C_{56}H_{72}N_{16}S_8Zn$+3H_2O: C 50.00, H 5.84, N 16.66%. Found: C 49.92, H 5.86, N 16.27%. Data from ref. [1]

UV–vis (pyridine) *λ*/nm (ε/$M^{-1}cm^{-1}$): 657 (298 000), 595 (33 600), 385 (146 000). ^{13}C NMR (75.43 MHz, $CDCl_3$) δ/ppm = 30.6, 51.2, 143.9, 150.6, 158.1. ^{1}H NMR (300 MHz, $CDCl_3$) δ/ppm = 1.90 (s, 72H, CH_3). Data from ref. [2]

2,3,9,10,16,17,23,24-Octakis(*tert*-butylsulfanyl)-1,4,8,11,15,18,22,25-(octaaza)phthalocyanine (**TPyzPzH2**)

Rf (toluene/chloroform 1:1) = 0.65. MS (MALDI-TOF) *m*/*z*: 1226.2 $[M]^+$, 1249.2 $[M^+Na]^+$, 1265.1 $[M^+K]^+$, 2452.4 $[2M]^+$, 2457.4 $[2M^+Na]^+$, 2491.3 $[2M^+K]^+$. Elemental analysis calculated for $C_{56}H_{74}N_{16}S_8$: C 54.78, H 6.07, N 18.25%. Found: C 54.63, H 6.17, N 17.89%. Data from ref. [1]

UV–vis (pyridine) *λ*/nm (ε/$M^{-1}cm^{-1}$): 656 (228200), 595 (30400), 384 (128200), (chloroform): 675 (228700), 642 (172500), 590 (30500), 482 (65700), 366 (147900). ^{13}C NMR (75.43 MHz, $CDCl_3$) *δ* 30.5, 51.6, 142.3, 146.5, 159.0. ^{1}H NMR (300 MHz, $CDCl_3$) *δ* 2.19 (s, 72H, CH_3). Data from ref. [2]

2,3,9,10,16,17,23,24-Octakis(*tert*-butylsulfanyl)phthalocyaninato Magnesium(II) (**PcMg**)

Rf (toluene/chloroform/THF 10:10:1) = 0.59. MS (MALDI-TOF) *m*/*z*: 1240.2 $[M]^+$, 2480.4 $[2M]^+$. UV-vis (DMF) *λ*/nm (ε/$M^{-1}cm^{-1}$): 700 (269 800), 631 (46 300), 368 (99 600). Elemental analysis calculated for $C_{64}H_{80}MgN_8S_8$+1H_2O: C 61.00, H 6.56, N 8.89%. Found: C 60.86, H 6.87, N 8.77%. Data from ref. [1]

UV–vis (pyridine) *λ*/nm (ε/$M^{-1}cm^{-1}$): 705 (275 000), 636 (48 800), 371 (93 400). ^{13}C NMR (75.43 MHz, CDCl3) δ/ppm = 137.5, 130.5, 128.3, 124.7, 48.6, 31.4. ^{1}H NMR (300 MHz, CDCl3) δ/ppm = 7.97 (s, 8H, Ar–H), 1.45 (s, 72H, CH3). Data from ref. [2]

2,3,9,10,16,17,23,24-Octakis(*tert*-butylsulfanyl)phthalocyaninato Zinc(II) (**PcZn**)

Rf (toluene/chloroform/THF 10:10:1) = 0.80. MS (MALDI-TOF) *m*/*z*: 1280.1 $[M]^+$, 2560.2 $[2M]^+$. UV-vis (DMF) *λ*/nm (ε/$M^{-1}cm^{-1}$): 700 (271 300), 630 (45 800), 369 (80 800). Elemental analysis calculated for $C_{64}H_{80}N_8S_8Zn$+1H_2O: C 59.07, H 6.35, N 8.61%. Found: C 59.04, H 6.59, N 8.43%. Data from ref. [1]

UV–vis (pyridine) *λ*/nm (ε/$M^{-1}cm^{-1}$): 692 (273 400), 625 (44 700), 360 (83 500). ^{13}C NMR(75.43 MHz, $CDCl_3$) δ/ppm = 135.7, 133.2, 130.1, 125.5, 48.6, 31.5. ^{1}H NMR (300 MHz, $CDCl_3$) δ/ppm = 6.98 (s, 8H, Ar–H), 1.43 (s, 72H, CH_3). Data from ref. [2]

2,3,9,10,16,17,23,24-Octakis(*tert*-butylsulfanyl)phthalocyanine (**PcH2**)
Rf could not be determined due to strong silica binding properties of this compound. The spot was always found on the start with Rf = 0. MS (MALDI-TOF) *m/z*: 1218.2 $[M]^+$. Elemental analysis calculated for $C_{64}H_{82}N_8S_8$+1H_2O: C 62.09, H 6.84, N 9.05%. Found: C 62.08, H 6.52, N 9.10%. Data from ref. [1]
UV−vis (pyridine) λ/nm (ε/$M^{-1}cm^{-1}$): 719 (119 500), 695 (119 100), 636 (29 000), 356 (64 700). ^{13}C NMR (75.43 MHz, $CDCl_3$) δ/ppm = 139.0, 130.0, 128.2, 125.5, 48.9, 31.4. ^{1}H NMR(300 MHz, $CDCl_3$) δ/ppm = 8.10 (s, 8H, Ar–H), 1.43 (s, 72H, −CH_3). Data from ref. [2]

2,3,9,10,16,17,23,24-Octakis(2-(triethylammonio)ethylsulfanyl)-1,4,8,11,15,18,22,25-(octaaza)phthalocyaninato Magnesium(II) Octaiodide (**ws-TPyzPzMg**).
UV−vis (DMF) λ/nm (ε/$M^{-1}cm^{-1}$): 655 (171 200), 596 (24 900), 386 (106 600). UV−vis (water) λ/nm (ε/$M^{-1}cm^{-1}$): 652 (166 300), 593 (23 800), 382 (114 600), 224 (132 700). ^{13}C NMR (75 MHz, D_2O + dimethylsulfoxide-d6): δ/ppm = 55.9, 53.5, 31.0, 7.8, aromatic signals were not detected. ^{1}H NMR (300 MHz, D_2O + dimethylsulfoxide-d6): δ/ppm = 4.45−4.27 (m, 16H, SCH_2), 3.90−3.73 (m, 16H, NCH_2), 3.70−3.52 (m, 48H, NCH_2), 2.36−2.14 (m, 72H, CH_3); all signals were broad. IR (ATR): 2978, 1658, 1519, 1452, 1396, 1334, 1309, 1250, 1178, 1108, 1093, 1027, 971, 852 cm^{-1}. Elemental analysis calculated for $C_{88}H_{152}I_8MgN_{24}S_8$ + 11H_2O: C 34.76, H 5.77, N 11.06%. Found: C, 34.75, H 5.45, N 10.68%. Data from ref. [1]

2,3,9,10,16,17,23,24-Octakis(2-(triethylammonio)ethylsulfanyl) tetrapyrazinoporphyrazine Zinc(II) Octaiodide (**ws-TPyzPzZn**)
UV−Vis (H_2O) λ/nm (ε/$M^{-1}cm^{-1}$): 652 (169 000), 592 (25 000), 376 (112 000). ^{13}C NMR (75.43 MHz, D_2O+C_5D_5N) δ/ppm = 55.6, 53.2, 30.0, 7.4 (aromatic signals not detected). ^{1}H NMR (300 MHz, D_2O+C_5D_5N) δ/ppm = 4.74–4.50 (broad, 16H, S-CH_2), 4.20–3.96 (broad, 16H, N-CH_2), 3.94–3.66 (broad, 48H, N-CH_2), 1.82–1.54 (broad, 72H, CH_3). IR (KBr) t = 2976, 1728, 1658, 1517, 1452, 1395, 1304, 1250, 1175, 1108, 1094, 971. Elemental analysis calculated for $C_{88}H_{152}I_8N_{24}S_8Zn$+3$H_2O$: C 35.98, H 5.42, N 11.44 % Found: C 36.17, H 5.52, N 11.20 %. Data from ref. [3]

2,3,9,10,16,17,23,24-Octakis[2-(triethylammonio) ethylsulfanyl]phtalocyaninato Magnesium(II) Octaiodide (**ws-PcMg**).
UV−Vis (DMF) λ/nm (ε/$M^{-1}cm^{-1}$): 702 (210 000), 629 (36900), 377 (91 400). UV−Vis (H2O) λ/nm (ε/$M^{-1}cm^{-1}$): 697 (68 000), 662 (88 600), 356 (77 100), 225 (150 900). ^{13}C NMR (125 MHz, CD_3OD) δ/ppm = 56.1, 53.7, 28.9, 7.4, signals of aromatic carbons were not detected. ^{1}H NMR (500 MHz, CD_3OD) δ/ppm = 9.47 – 9.32 (br, 8 H, ArH), 4.66 – 3.38 (br, 80 H, SCH_2 + NCH_2), 1.66 – 1.09 (m, 72 H, CH_3). IR (ATR): ν = 3433, 2977, 1595, 1471, 1454, 1402, 1371, 1283, 1186, 1155, 1111, 1067, 1022, 942 cm^{-1}. Elemental analysis calculated for $C_{96}H_{160}I_8MgN_{16}S_8$ + 6H_2O: C 39.18, H 5.89, N 7.62 %. Found: C 39.21, H 5.63, N 7.71 %. Data from ref. [4]

2,3,9,10,16,17,23,24-Octakis[2-(triethylammonio)ethylsulfanyl]phtalocyanine Octaiodide (**ws-PcH2**).
UV−Vis (DMF) λ/nm (ε/$M^{-1}cm^{-1}$): 700 (201 500), 629 (37 700), 371 (75 900). UV−Vis (H2O) λ/nm (ε/$M^{-1}cm^{-1}$): 668 (76 800), 431 (sh), 341 (60 700), 224 (147 100). ^{13}C NMR (75 MHz, CD_3OD) δ/ppm = 56.1, 53.7, 7.4, one signal of aliphatic carbon and signals of aromatic carbons were not detected. ^{1}H NMR (300 MHz, CD_3OD) δ/ppm = 9.50 (s, 8 H, ArH), 4.41 – 3.96 (br, 16 H, SCH_2), 3.94 – 3.79 (br, 16 H, NCH_2), 3.78 – 3.47 (br, 48 H, NCH_2), 1.54 – 1.14 (m, 72H, CH_3). IR (ATR): ν = 3439, 3290, 2977, 1597, 1453, 1418, 1399, 1367, 1286, 1155, 1135, 1077, 1021, 935 cm^{-1}. Elemental analysis calculated for $C_{96}H_{162}I_8N_{16}S_8$ + 6H_2O: C 39.48, H 6.01, N 7.67 %. Found: C 39.24, H 5.63, N 7.69 %. Data from ref. [4]

2,3,9,10,16,17,23,24-Octakis[2-(triethylammonio)ethylsulfanyl]phtalocyaninato Zinc(II) Octaiodide (**ws-PcZn**).

UV–Vis (H_2O) λ/nm (ε/$M^{-1}cm^{-1}$): 699 (sh), 660 (100 000), 360 (80 000), 226 (157 200). UV–Vis (DMF) λ/nm (ε/$M^{-1}cm^{-1}$): 704 (280 300), 631 (48 000), 384 (103 000). ^{13}C NMR (125 MHz, CD_3OD) δ/ppm = 57.3, 54.8, 29.0, 8.7, the signals of aromatic carbons were not detected. ^{1}H NMR (500 MHz, CD_3OD) δ/ppm = 9.37 (s, 8 H, ArH), 4.35 – 3.91 (br, 16 H, SCH_2), 3.91 – 3.79 (br, 16 H, NCH_2), 3.79 – 3.48 (br, 48 H, NCH_2), 1.52 – 1.14 (m, 72 H, CH_3). IR (ATR): ν = 3437, 2977, 1594, 1484, 1454, 1403, 1372, 1282, 1186, 1155, 1114, 1088, 1068, 943 cm^{-1}. Elemental analysis calculated for $C_{96}H_{160}I_8N_{16}S_8Zn$ + $4H_2O$: C 39.12, H 5.74, N 7.60 %. Found: C 39.09, H 5.70, N 7.63 %. Data from ref. [4]

References

1. Zimcik, P.; Novakova, V.; Kopecky, K.; Miletin, M.; Uslu Kobak, R.Z.; Svandrlikova, E.; Váchová, L.; Lang, K. Magnesium Azaphthalocyanines: An Emerging Family of Excellent Red-Emitting Fluorophores. *Inorg. Chem.* **2012**, *51*, 4215-4223. doi: 10.1021/ic2027016
2. Kostka, M.; Zimcik, P.; Miletin, M.; Klemera, P.; Kopecky, K.; Musil, Z. Comparison of aggregation properties and photodynamic activity of phthalocyanines and azaphthalocyanines. *J. Photochem. Photobiol., A* **2006**, *178*, 16-25. doi:
3. Zimcik, P.; Miletin, M.; Radilova, H.; Novakova, V.; Kopecky, K.; Svec, J.; Rudolf, E. Synthesis, Properties and In Vitro Photodynamic Activity of Water-soluble Azaphthalocyanines and Azanaphthalocyanines. *Photochem. Photobiol.* **2010**, *86*, 168-175. doi: 10.1111/j.1751-1097.2009.00647.x
4. Machacek, M.; Cidlina, A.; Novakova, V.; Svec, J.; Rudof, E.; Miletin, M.; Kucera, R.; Simunek, T.; Zimcik, P. Far-Red-Absorbing Cationic Phthalocyanine Photosensitizers: Synthesis and Evaluation of the Photodynamic Anticancer Activity and the Mode of Cell Death Induction. *J. Med. Chem.* **2015**, *58*, 1736-1749. doi: 10.1021/jm5014852
